# Spatio-temporal soil loss modelling using RUSLE and sediment delivery into a reservoir in a semi-arid region of northern Nigeria

**DOI:** 10.1016/j.heliyon.2024.e38887

**Published:** 2024-10-05

**Authors:** Ali Aldrees, Samaila Jibrin El-pateh, Salisu Dan'azumi, Sani Isah Abba

**Affiliations:** a Department of Civil Engineering, College of Engineering in Al-Kharj, Prince Sattam Bin Abdulaziz University, Al-Kharj, 11942, Saudi Arabia; b Department of Civil Engineering, Faculty of Engineering, Bayero University Kano, 700241, Kano, Nigeria; c Department of Chemical Engineering, Prince Mohammad Bin Fahd University, Al Khobar, 31952, Saudi Arabia; d Water Research Center, Prince Mohammad Bin Fahd University, Al Khobar, 31952, Saudi Arabia

**Keywords:** Erosion, Sedimentation, GIS, RUSLE, Bauchi, Nigeria

## Abstract

Soil loss is aggravated by uncontrolled deforestation, indiscriminate land clearing for agricultural activities, overgrazing, and urban development that leads to severe soil erosion over the land surface. The main objective of this research is to apply the Revised Universal Soil Loss Equation (RUSLE), in conjunction with remote sensing and GIS, to determine the temporal variation of soil loss from the Gubi watershed in the years 2000 and 2017 and to estimate the sediment delivery into the Gubi reservoir in Northern Nigeria. Datasets of rainfall, soil type, topography, cover management, and support practice were utilized to determine the five RUSLE factors. The research identified five levels of erosion ranging from severe to very low-risk areas. The spatial distribution of erosion hotspots in the catchment area was highlighted. Results revealed increasing levels of erosion from 14,244.31 tons/year in 2000 to 16,792.33 tons/year in 2017. Consequently, the catchment's sediment yield was 6903.65 tons/year and 9172.71 tons/year corresponding to sediment delivery ratios of 48.46 % and 54.62 % in 2000 and 2017 respectively. The sediment inflow into Gubi reservoir was 747.38 tons/year and 950.76 tons/year corresponding to 10.83 % and 10.37 % of the total sediment yield in 2000 and 2017 respectively. Soil erosion vulnerability index maps were produced and the erosion is expected to increase with time as agricultural activities and deforestation continue to occur. The sediment deposits have the potential to reduce the designed life of the reservoir. Best management practices, such as tree planting, mulching, and contour ridging are recommended for soil conservation. There is a need to assess the reservoir sediment deposits so that measures can be taken to maintain the reservoir operations properly.

## 1 Introduction

Soil erosion is one of the environmental problems facing the world in the 21st century, upsetting human society and universally recognized as a serious hazard to the well-being of humans [1,2]. It is considered the second environmental problem the world suffers from, after population growth [3,4]. Soil loss is mostly caused by agricultural activities, which lead to altered forests with consequent diversity losses [5,6]. A study by Wardafo [7] showed that over eighty percent of the world's expanse of agricultural land suffers from moderate to harsh erosion, which leads to the loss of productivity. Coupled with the population growth, this leads to the present fall in the global per capita food supply.

Soil erosion is the process in which the surface layer of the weathered rock is loosened and carried away by running water or wind [3,8]. The most dominant agent of erosion is water and the process includes detachment, transportation, and deposition of particles (sediment) by impact of raindrops and flowing water [9]. Erosion usually occurs when soil is left exposed to rain or wind energy, where raindrops hit exposed soil with some energy and displace the soil particles [10]. Studies have discovered that eroded soils are one of the main problems in agriculture and natural resources management. Erosion reduces soil efficiency, fills reservoirs, and pollutes the streams [[11], [12], [13], [14]]. The resultant effect is a decline in landscape beauty, a threat to food security, an increase in the probability of flood in flood plains, loss of aquatic biodiversity in rivers and lakes, reduced water quality by pollution, and eutrophication [[15], [16], [17]]. Gubi catchment undergoes some dramatic changes in land-use-land-cover (LULC) due to urbanization. These changes caused an increase in soil loss affecting the water quality of the streams entering the reservoir. The sediment-laden water ultimately finds its way into the reservoir leading to reservoir sedimentation.

Several models have been developed to estimate sediment yields such as the European soil erosion model (EUROSEM), Chemicals runoff and erosion from agricultural management system (CREAMS), Erosion-productivity impact models (EPI) Universal soil loss equation (USLE), Soil loss estimation model for Southern Africa (SLEMSA) and Revised universal soil loss equation (RUSLE). USLE has emerged as a leading model that has been broadly used all over the world in both agricultural and hilly watersheds owing to its simplicity of obtaining parameters [8]. The USLE was improved and replaced by the RUSLE. RUSLE is widely used and is meant to overcome the limitations of the USLE, but the model still inherits its basic properties from the USLE [[18], [19], [20]].

Over the years, many researchers have used RUSLE to quantify soil loss in various watersheds worldwide. Kouli et al. [21] used RUSLE and GIS to predict soil loss in the Crete watershed (Greece) and a high correlation between steep slopes and poor surface cover was found. Shi et al. [22] categorized soil loss into classes within the Wangjiaqiao watershed (China). About 26 tons/ha of soil loss was found to be from the flatter agricultural areas while 52 tons/ha represented cultivation occurring on steep lands, the latter being the major contributor to sediment transport in the watershed. Marondedze and Schütt [23] used RUSLE to predict potential soil loss in the Epworth District (Zimbabwe). Results revealed that urbanization results in soil erosion risk which threatens about 40 % of the Epworth district. Bou-imajjane and Belfoul [24] indicated an average annual soil loss of 40.38 t/ha/year in the Beni Mohand River Basin (Morocco). The basin was shown to be subjected to very high rates of erosion which could be irreversible. RUSLE is the most widely used model for soil loss estimation, all over the world. A literature review of the global application of USLE and RUSLE indicated that over 1556 researches were conducted between 1960 and 2021 [25].

GIS application in soil erosion modeling is increasing because of the advantages of combining it with soil erosion models. This is useful because it allows the simulation of large-scale studies using large amounts of data requiring relatively a short processing time. Secondly, GIS also permits the simulation of different scenarios from various changing land-use conditions and management alternatives in space and time [[26], [27], [28], [29], [30], [31]]. The combination of GIS with erosion models such as the RUSLE has improved the efficiency for estimating spatial distribution and magnitude of erosion risk with reasonable costs and better accuracy [[32], [33], [34], [35]].

Soil erosion leads to land degradation. The eroded soil eventually finds its way to receiving water bodies leading to reservoir sedimentation. Sediment yield is dependent on gross erosion in the watershed and on the transport of eroded material out of the watershed. Only a part of eroded material from upland areas in a watershed is carried out of the watershed [36,37]. Between the source and the outlet, varying proportions of the eroded materials are deposited, for example, particles eroded from bare upland areas may be trapped in vegetated areas, lakes, or reservoirs. The total amount of sediment that is delivered to the outlet of the watershed is known as the sediment yield [38]. Daramola et al. [39] used the SWAT model to assess the sediment yield from Kaduna River Basin (Nigeria) into the Shiroro reservoir from 1990 to 2018. Results showed that an estimated suspended sediment yield of about 84.1 tons/ha/yr was deposited within the period under study. Bihonegn and Awoke [40] assessed the impact of LULC on the sediment yield of Koka Reservoir (Ethiopia) in 2005, 2010, and 2015. Results showed an increasing sediment yield from 26.03 tons/ha/yr to 26.34 tons/ha/yr to 28.33 tons/ha/yr for 2005, 2010, and 2015 respectively. Tan et al. [41] used four empirical trap efficiency models proposed by Brune, Brown, Siyam, and Jothiprakash to determine the improved trap efficiency of large reservoirs on the Yangtze River. Results showed that Brune and Siyam's models gave the best results. Reservoir sedimentation issues have attracted a lot of attention and many works have been carried out. However, data on soil loss and sediment delivery is generally lacking in sub-Saharan Africa and the Gubi watershed is one of those catchments facing this issue. The aim of this research is to use GIS and RUSLE to determine the soil loss rate for two time periods (2000 and 2017) from the Gubi catchment and to estimate the sediment delivery into the Gubi reservoir.

## 2 Materials and methods

### 2.1 Study area

Gubi catchment is located in the southern part of Bauchi State, North-Eastern Nigeria. It is a tributary to the Gongola River which flows from Jos Plateau, southwest of Bauchi State to the northeast. It has a catchment area occupying approximately 17,921 ha comprising 5 major sub-catchments. The prevailing climate is semi-arid tropical, having a mean annual rainfall of 1200 mm. The rainy season lasts for 5 months and normally starts at the end of April and increases in successive months until August when the rain reaches its peak and the season ends in early October [42]. The temperature regime is warm to hot for most of the year, with a slightly cool period between November and February. The mean maximum monthly temperature is 38 °C occurring in March and April, the minimum monthly temperature is 10 °C in December and January. The relative humidity rises to 75 % during the rainy months and drops to less than 10 % during the dry season, especially in the harmattan period, from mid-November to mid-February [42]. The major soils in and around the study area are Lithic Leptosols, Undifferentiated Luvisols, Dystric Nitrosols, Gleyic Luvisols, and Plinthic Lixisols [43,44].

### 2.2 Digital Elevation Model

**Fig. 1:**
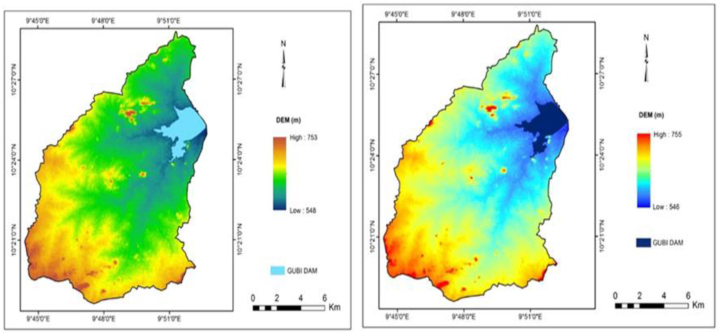
a: DEM of Gubi catchment (SRTM, 2000) Fig. 1. b: DEM of Gubi catchment (Alaska DEM, 2015).

Digital Elevation Model (DEM) for the study area, in the form of Shuttle Radar Topography Mission (SRTM) 2000 and Alaska DEM 2015, were obtained from the Earth Explorer website (earthexplorer.usgs.gov) and (www.asf.alaska.edu/#) respectively (Fig. 1a and b). the DEM represents the terrain as a continuous surface of the catchment and permits retrieval of geographical information that can be used to identify different basin characteristics.

### 2.3 Revised Universal Soil Loss Equation (RUSLE)

The methodology was based on the principle of the RUSLE. The method involves the isolation of each variable and quantifying its erosional effect such that the product of all the variables gives the amount of soil loss in the field concerned. The analysis was carried out in the ArcGIS. The RUSLE modeling used available rainfall data, soil properties, topography, vegetation, and land use. Table 1 shows the RUSLE factors, type, and sources of input data and their spatial resolution.

**Table 1:** Sources of data for RUSLE input parameters.

S/N	RUSLE Factor	Type of Data	Data Sources	Spatial resolution
1	Rainfall erosivity factor (R)	Monthly rainfall data	i. Bauchi State Agricultural Development Project, Zonal Office, Bauchi (1995–2017)	10.94°N, 9.81^o^E
			ii. Bauchi Old Airport Station, Nigerian Metrological Agency (NIMET) (1990–2017)	10.30°N, 9.82^o^E
			iii. College of Agriculture, Bauchi (1998–2017)	10.28°N, 9.79^o^E
			iv. School General Studies, Abubakar Tatari Ali Polytechnic, Bauchi, (2009–2016)	10.31°N, 9.77^o^E
			v. NIMET New Airport, Bauchi, (2011–2017) (Supplementary File (Tables S1a–S1e)	10.48°N, 9.75^o^E
2	Soil erodibility factor (K)	Soil samples	Fifteen (15) locations within the Gubi catchment 17,921 ha (Fig. 2) (Supplementary File, Tables S2a–S2d)	1 soil sample per 1195 ha (See Fig. 2)
3	Slope length and steepness factor (LS)	DEM data	i. SRTM (earthexplorer.usgs.gov)	30m × 30m
			ii. Alaska DEM (www.asf.alaska.edu/#)	12.5m × 12.5m
4	Cover management factor (C)	LANDSAT7 ETM + Image data (2000) LANDSAT8 OLI (2017)	i. Global Land Cover Facility (GLCF) https://geog.umd.edu/feature/global-land-cover-facility-%28glcf%29	30m × 30m 30m × 30m
			ii. United States Geological Survey (USGS) https://www.usgs.gov/centers/eros/science/usgs-eros-archive-landsat-archives-landsat-8-oli-operational-land-imager-and	
5	Conservation practice factor (P)	LANDSAT7 ETM + Image data (2000) LANDSAT8 OLI (2017)	i. Global Land Cover Facility (GLCF)https://geog.umd.edu/feature/global-land-cover-facility-%28glcf%29	30m × 30m
			ii. United States Geological Survey (USGS)https://www.usgs.gov/centers/eros/science/usgs-eros-archive-landsat-archives-landsat-8-oli-operational-land-imager-and	30m × 30m

The RUSLE equation (Equation (1)) was used to calculate the average annual soil loss. Determination of the RUSLE factors is presented in Sections 2.3.1 to 2.3.5.

(1) *A = R x K x LS x C x P*

where: A is the average annual soil loss per hectare (t ha^−1^ y^−1^),

R is the rainfall erosivity factor (MJ mmha^−1^h^−1^y^−1^).

K is the soil erodibity factor (t haMJ^−1^ mm^−1^).

LS is the slope-steepness factor (dimensionless).

C is the cover management factor (dimensionless).

P is the conservation practices factor (dimensionless).

#### 2.3.1 Rainfall erosivity factor (*R*)

Determination of the R-factor is best done with 30-min rainfall data, which is not available in Gubi as the catchment is located in sub-Saharan Africa where fine-resolution data is scarce. Therefore, monthly rainfall data for the study area was collected, from 5 meteorological stations nearby, for a period of 27, 22, 19, 8, and 6 years from the NIMET Old Airport, College of Agriculture Bauchi, Bauchi State Agricultural Development Project, Abubakar Tatari Ali Polytechnic and NIMET New Airport respectively. The R-factors for the periods were calculated from the mean annual rainfall using the formula proposed by Ref. [45] (Equations (2), (3))).

(2) $$\begin{array}{cc} R=0.04830\;P^{1.610} & P\;<\;850mm \end{array}$$

(3) $$\begin{array}{cc} R=587.7\;–\;1.219P+0.004105P^{2}\; & P\;>\;850mm \end{array}$$

where: *R* is the rainfall erosivity factor and *P* is the mean annual rainfall (mm).

The data was then interpolated in the GIS (ArcGIS) environment to produce continuous rainfall data for each grid cell using the spatial analyst tool.

#### 2.3.2 Soil erodibility factor (*K*)

**Fig. 2:**
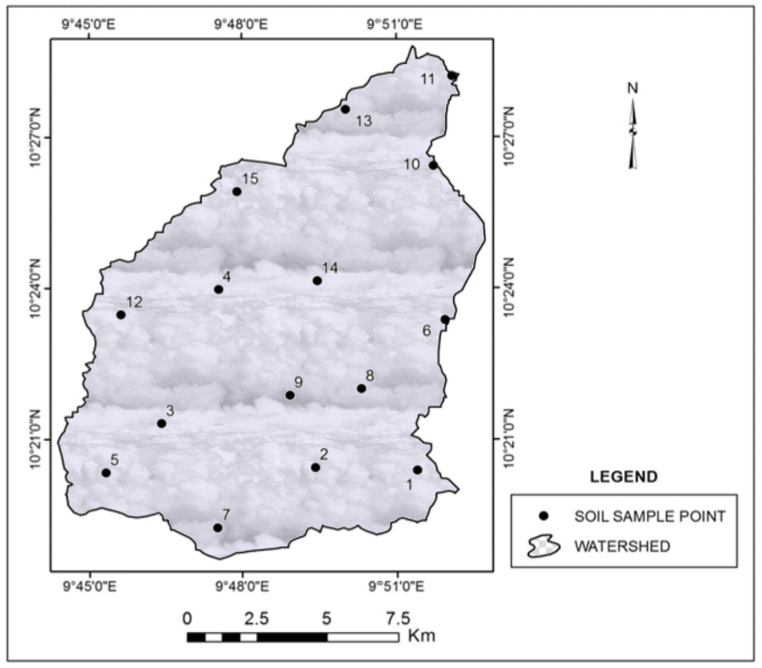
Location of soil sample collection points.

Fifteen (15) soil samples were collected within the catchment and taken to the laboratory for analysis (Fig. 2). For this study, soil texture, structure class, organic matter, and permeability class were considered and the K-factor was calculated (Equations (4), (5))) [46]. For soils with more than 70 % silt plus very fine sand, Equation (4) was used**.** For soils with less than 70 % silt, Equation (5) was used in the estimation of *K*.

(4) *K = 2.77 M*^*1.14*^*(10*^*−7*^*) (12 - OM) + 4.28(10*^*−3*^*) (St - 2) + 3.29(10*^*−3*^*) (Pt - 3)*

(5) *K= [2.1 x 10*^*˗4*^*x (12 - OM) x M*^*1.14*^*+ 3.25 x (St - 2) + 2.5 x (Pt - 3)]/100*

where: *OM* = Organic matter content (%)

*M* = (%Silt + fine sand) × (100- Clay).

*St* = Soil structure code.

*Pt* = Permeability class.

The laboratory results of the soil's particle size distribution, organic matter contents, structure code, and permeability class are presented in Supplementary File 3.

The shape file was added as a layer into ArcGIS, where the soil raster map was generated from the soil data using inverse distance weight. The soil map attribute table was edited and the values of *K* for the different soil types within the study area were selected and assigned the *K* factor. The interpolation in the ArcGIS spatial analyst tool was carried out to generate a continuous soil data map.

#### 2.3.3 Slope length and slope steepness factor (LS)

The data of topographic effects on erosion (*L* and *S*) were computed together using the available DEM data extracted from SRTM 30m resolution and Alaska DEM 12.5m resolution for two time periods: years 2000 and 2017. The *LS* factor was generated using Equation (6) [47].

(6) $$LS={\left(Flow\;accumulation\times {\left(\frac{Cell\;size}{22.13}\right)}^{0.4}\;\left(\frac{\sin\left(slope\right)}{0.0896}\right)\right)}^{1.3}$$

#### 2.3.4 Vegetation cover and management factor (C)

Satellite images from LANDSAT7 ETM+; GLCF for 2000 and LANDSAT8 OLI; USGS were used. Supervised classification of the LANDSAT image was done using ERDAS IMAGINE software, which was used to prepare the LULC map and the normalized difference vegetation index (NDVI) of the study area. The C-factor was selected based on the land cover and management factor values (Table 2).

**Table 2:** Cover and management factor values [48].

Code	Land Cover type	Cover Management Factor (C)	Land use
1	Water Bodies	0	Water
2	Bare Soil with Rock Outcrops	0.5	Barren
3	Developed	0.003	Built environment
**4**	Light vegetation	0.05	Forest
5	Thick forest	0.004	
6	Agriculture	0.3	Agriculture

#### 2.3.5 Support practices factor (P)

Satellite images from LANDSAT7 ETM+; GLCF for 2000 and LANDSAT8 OLI; USGS were used to obtain the land-use type and slope. There are no major conservation support practices being followed in the study area, except that the agricultural plots under cultivation are bunded. As data were lacking, the P-factor values corresponding to different land uses and slopes suggested by Ayele [49] were adopted as presented in Table 3.

**Table 3:** P-value of different land use types [49].

Land use type	Slope (%)	P-factor
Agricultural land	0–5	0.1
	5–10	0.12
	10–20	0.14
	20–30	0.19
	30–50	0.25
	50–100	0.33
Other land	All	1.0

#### 2.3.6 Computation of soil loss (A)

**Fig. 3:**
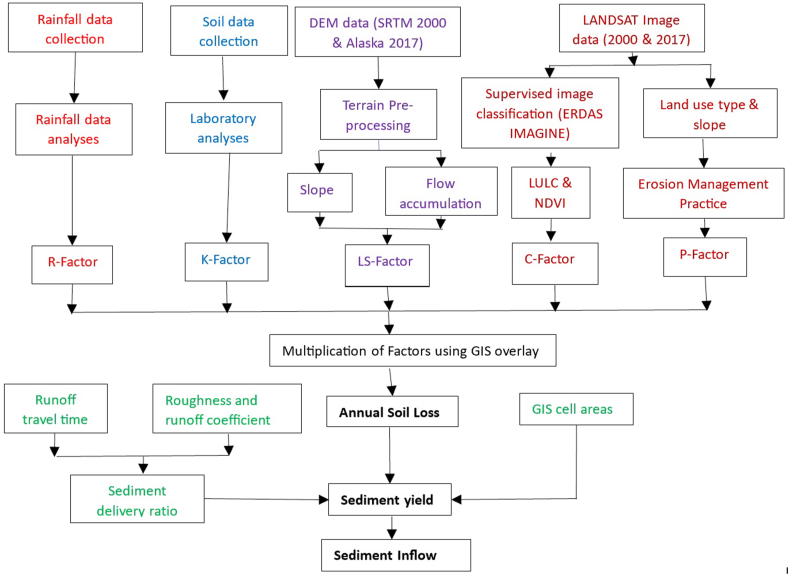
Integration of RUSLE with ArcGIS for the determination of soil loss and sediment transport.

Having computed the various RUSLE factors, the components were integrated into a GIS environment to simulate the action of erosion in the watershed and the annual amount of soil loss was estimated. As the soil type and average annual rainfall values for the two time periods are not expected to vary significantly, similar *K* and *R* factors were used for the years 2000 and 2017. However, variations are expected in the basin's land use and topography, thus, there is a need to determine the various land use maps and DEMs for the two periods. For each of the study periods, the *LS* and *C* factors were estimated and combined with the remaining RUSLE factors using the Raster calculator of ArcGIS to obtain the average annual soil loss (*A*) in tons/ha/year. The annual soil loss maps for two periods were produced. The soil losses were further classified into six different erosion risk levels (ie: severe, very high, high, moderate, low, and very low) [50]. The overall methodology that describes the integration of RUSLE into ArcGIS for the determination of the total annual soil loss is presented in Fig. 3.

### 2.4 Estimation of sediment inflow to Gubi reservoir

Some fraction of the eroded sediment from the Gubi catchment is transported into the reservoir. The sediment inflow was estimated from the sediment yield, which is the amount of sediment that enters the downstream reservoir. The method involves the calculation of the sediment delivery ratio for each morphological unit or grid cell using raster GIS from the runoff travel time and roughness coefficient (Fig. 3). The sediment yield for the entire Gubi watershed was calculated using Equation (7) [51].

(7) $$S_{y}=\sum_{i=1}^{n}\left({SDR}_{i}*{SL}_{i}\right)$$

where *SDR*_*i*_ is the sediment delivery ratio, *SLi* is the amount of soil erosion produced within the *ith* cell of the catchment which was estimated using the RUSLE equation and *n* is the total number of cells over the catchment.

Using ArcGIS, yield calculations were performed for each grid cell and then summed across the watershed.

#### 2.4.1 Sediment delivery ratio (*SDR*)

The *SDR*_*i*_ of each pixel (cell) is the fraction of the gross soil loss from *cell i* that reaches a continuous stream system and is defined as Equation (8) [52].

(8) *SDR*_*i*_*=* exp *(-β ∗t)*

Where: *t* = runoff travel time from *ith* cell to nearest stream reach (hr).

*β* = roughness and runoff coefficient for *cell i* (dimensionless).

##### 2.4.1.1 Travel time

Runoff travel time (*t*_*i*_*)* is the time taken for runoff to travel from *cell i* to the stream network and was calculated using Equation (9) [51,53].

(9) $$t_{i}=\sum_{i=1}^{n}\left(\frac{l_{i}}{v_{i}}\right)$$

where: *li* is the flow length of *cell i* which is the length of a square side or to a diagonal depending on the direction of flow in the ith cell. *v*_*i*_ is the velocity of flow in *cell i* and is considered to be a function of the land surface slope and the land cover characteristics, thus *v*_*i*_ is defined by Equation (10) [54].

(10) $$v_{i}=a_{i}\;*\;{S_{i}}^{0.5}$$

where *S*_*i*_ is the slope of the *ith* cell and *a*_*i*_ is a coefficient related to land use. The values of *ai* were adopted from Ref. [55]. The travel time was computed to the nearest channel in the flow path direction and finally to the dam inlets.

##### 2.4.1.2 Roughness and runoff coefficient for *cell i*

*β* Is the basin-specific coefficient which depends on the watershed's morphological data. It was estimated using Equation (11) [56].

(11) $$\beta =-\frac{\ln\;\left({SDR}_{w}\right)}{1000*a}$$

where *SDR*_*w*_ is the watershed's sediment delivery ratio and *a* is an estimated coefficient depicting the sediment transport efficiency. They were calculated using Equations (12), (13)) respectively [56].

(12) $${SDR}_{w}=0.4724*\;U^{-0.125}$$

(13) $$a=0.00111t_{m}$$

where *t*_*m*_ = mean travel time which was computed from the attribute table of the travel time map as 3.914 h (Figs. S4a and S4b under Supplementary File 4).

### 2.5 Model validation

The sediment yield of the Gubi catchment was compared with measured sediments from six catchments in Northern Nigeria to validate the result. Furthermore, the sediment yield estimated by RUSLE was compared with those computed using an empirical equation proposed by Fournier (Equation (14)) [57].

(14) $$\mathrm{Log}\;Q_{si}=2.65\;\mathrm{Log}\frac{P_{w}^{2}}{P_{a}}+0.46LogH\left(tanS\right)-1.56$$

where *Q*_*S*_ is the annual sediment yield (tons/km^2^/yr), which was converted to tons/ha/yr, *P*_*W*_ is the mean rainfall depth during the rainiest month (mm), *P*_*a*_ is the annual rainfall depth (mm), *H* is the average height of the watershed (m), and *S* is the average slope of the watershed (degree).

## 3 Results

### 3.1 Rainfall erosivity factor (*R*)

**Fig. 4:**
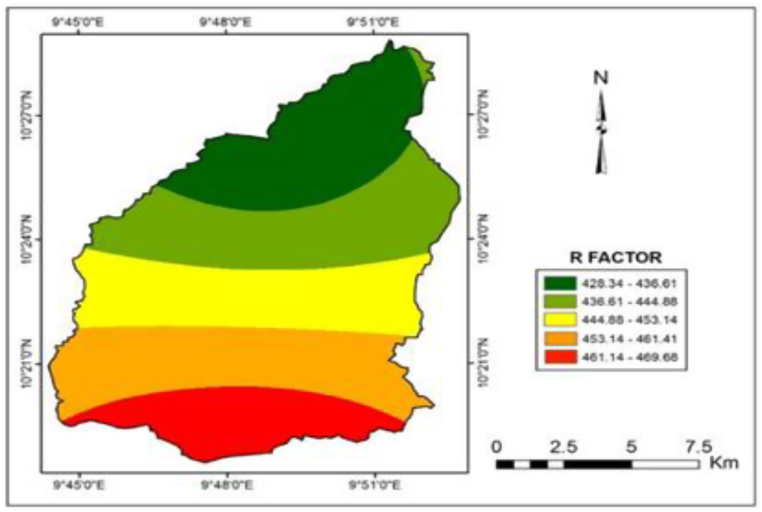
Iso-erodent map showing R factor (MJ.mm/ha.hr.year).

This is the erosive potential of rainfall. The rainfall erosivity map shows that the R-factor ranges from 428.34 to 469.68 MJ mm/ha/yr over the entire area. It is quite evident from the iso-erodent map (Fig. 4), that there was slight rainfall variability along the basin, where the southern and central parts have more rainfall compared with the northern part. The total erosive power in the southern part of the catchment is higher compared with the northern part. This spatial variation also implies that soil loss varies between rainstorm events.

### 3.2 Soil erodibility factor (*K*)

**Fig. 5:**
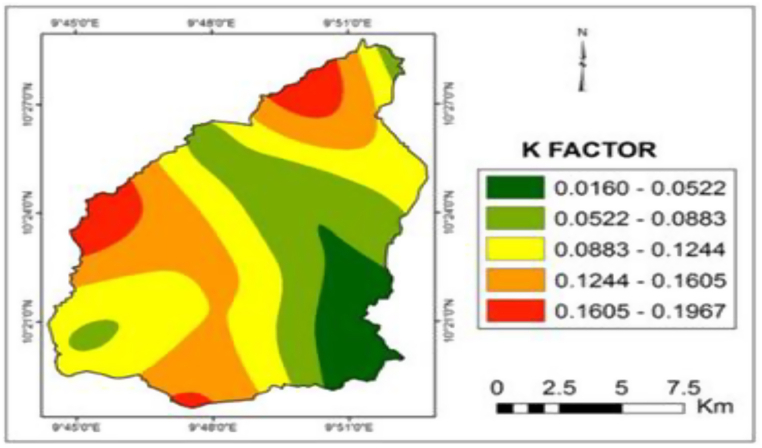
K-factor map of Gubi catchment.

The soil erodibility factor map (Fig. 5) was prepared from soil samples of the study area based on different soil textures. Generally, the majority of field soil samples show loamy sand fractions which comprise of a mixture of sand and clay particles with a high proportion of organic matter, except the southwestern part of the basin which is sandy.

The results indicated that K-Factor in the study area ranged from 0.0160 to 0.1967 tons.ha/MJ.mm. According to Lorentz and Schulze [50], the erodibility rating of the study area may be considered to fall under very low. The low K-factor was due to the presence of clay and high organic matter content in the soil, which provide resistance to erosion and the presence of coarse grain particles which reduces the erosion rate because greater force is required to entrain them.

### 3.3 Slope length and slope steepness (*LS*)

**Fig. 6:**
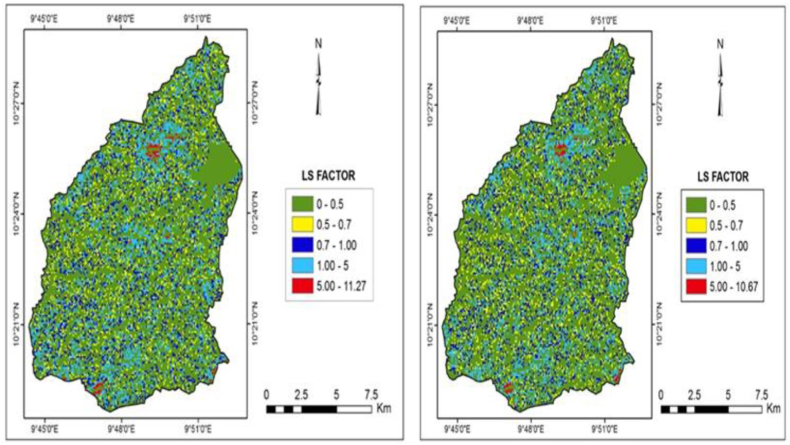
a: LS Factor Map of (2000) Fig. 6. b: LS Factor Map of (2017).

The LS-factor surface map is shown in Fig. 6a and b, with values ranging from 0 to 11.27° in the year 2000 and 0 to 10.67° in 2017. Thus, the variation of the LS-factor is mild. From the reclassified class intervals, the majority of the area (85.47 % and 87.34 %) falls below 1°. Most of the high values are noticed along small portions of the areas bordering the south, southeast, and northeast.

### 3.4 Crop management factor (*C*)

**Fig. 7:**
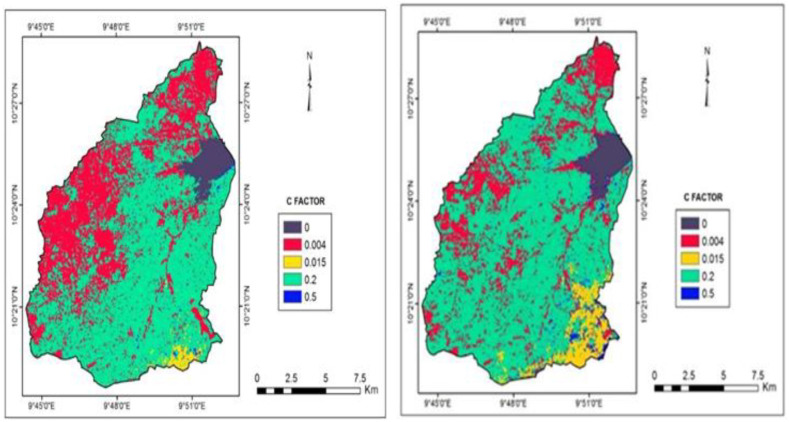
a: C-factor map for the year 2000 Fig. 7. b: C-factor map for the year 2017.

The C-factor maps in Fig. 7a and b depict that the natural vegetation condition is well reflected, and has a positive relationship with soil loss in the basin. Another important characteristic of the map is the wide variation of the C-factor with its non-uniformity in spatial distribution within the two periods. The land use of the watershed has changed substantially over the time frame, leading to major soil erosion problems. The forest area was rapidly deforested (legally or illegally) and substituted with agriculture, urbanization, and infrastructure development; all of which have contributed to severe soil erosion. Over the 17 years, the forest area was reduced from 32.65 % in 2000 to 21.30 % in 2017, leaving the land exposed, and posing huge erosion risk.

Table 4 shows the distribution of LULC for Gubi watershed in 2000 and 2017. The largest contributor to soil erosion in the watershed is agricultural activities, which cover a total land area of 107.08 km^2^ (59.75 % area) in the year 2000 and increased to 121.26 km^2^ (67.66 % area) in 2017. In addition, the built-up area significantly contributed to soil erosion in the basin, which included new settlements, shops, and hotels, which increased from 1.91 % of the area in the year 2000 to 3.19 % in 2017. The possible explanation for this is the aggravated anthropogenic factors such as population growth and urban migration that lead to the creation of new settlements. Deforestation, overgrazing, and agricultural practices have also increased tremendously in the basin, resulting in higher soil loss since 2000.

**Table 4:** LULC in the Gubi catchment.

Land Use Classification for 2000				Land Use Classification for 2017			
LULC	C-factor	Area (km^2^)	Area (%)	**LULC**	C-factor	Area (km^2^)	Area (%)
Waterbody	0	6.79	3.79	**Waterbody**	0	6.81	3.80
Vegetation	0.004	58.51	32.65	**Vegetation**	0.004	38.17	21.30
Built-up area	0.015	3.423	1.91	**Built-up area**	0.015	5.72	3.19
Agriculture	0.20	107.08	59.75	**Agriculture**	0.20	121.26	67.66
Bare surface	0.50	3.401	1.90	**Bare surface**	0.50	7.26	4.05
TOTAL		179.21	100.00	**TOTAL**		179.21	100.00

### 3.5 Soil management practice factor (*P*)

**Fig. 8:**
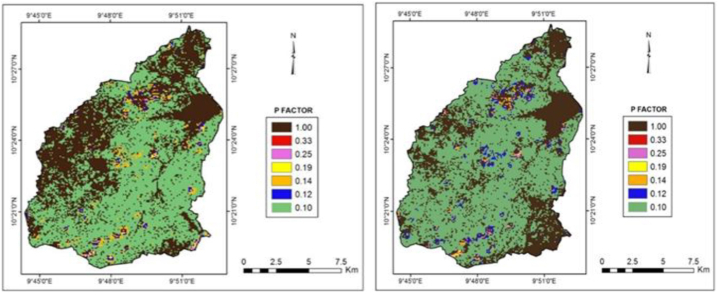
a: P-factor map for the year 2000 Fig. 8. b: P-factor map for the year 2017.

Fig. 8a and b presents the P-factor maps of the study area. The results depict that most parts of the area were highly vulnerable as most farming activities were carried out without any conservation practice. Table 5 presents the values of the support practice factor (*P*) according to the agricultural land and slope of the Gubi watershed.

**Table 5:** P-factor of different land use types [49].

P-Factor Classification for 2000				P-Factor Classification for 2017			
Slope Class (%)	P-value	Area (km^2^)	Area (%)	**Slope Class (%)**	P-value	Area (km^2^)	Area (%)
0–5	0.1	100.361	0.02	**0–5**	0.1	114.71	114.708
5–10	0.12	2.03	0.24	**5–10**	0.12	4.91	4.91
10–20	0.14	4.77	0.12	**10–20**	0.14	1.75	1.75
20–30	0.19	0.58	0.98	**20–30**	0.19	0.21	0.21
30–50	0.25	0.29	2.74	**30–50**	0.25	0.42	0.42
>50	0.33	0.02	64.01	**>50**	0.33	0.04	0.04
Other land	1	71.16	31.90	**Other land**	1	57.17	57.17
Total	–	179.21	100.00	**Total**	–	179.21	

### 3.6 Total annual soil loss (*A*)

**Fig. 9:**
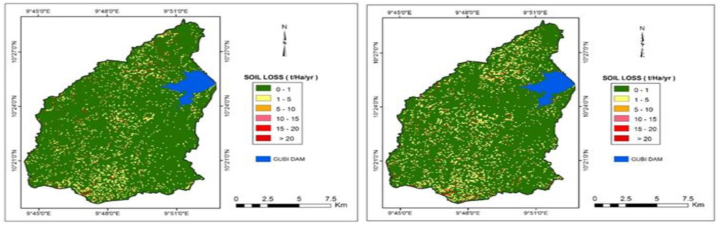
a: Soil erosion map for 2000 Fig. 9. b: Soil erosion map for 2017.

Annual soil loss maps for the catchment area were produced for the years 2000 and 2017 (Fig. 9a and b). The Gubi catchment in 2017 showed an increase in high, very high to severe erosion areas compared to the year 2000. The soil loss ranged from 0.15 to 43.75 tons/ha/year in 2000 and 0.19–43.59 tons/ha/year in 2017 (Table 6, Table 7) with corresponding mean soil losses of 0.80 tons/ha/year in 2000 and 0.94 tons/ha/year in 2017. The land use of Gubi catchment has changed substantially over this time frame leading to the increasing trend in major soil erosion problems from 2000 to 2017. Looking at the spatial distribution of soil loss between the two study periods, most of the steep areas of the southwestern part, central and the northeastern escarpments have high erosion potential. The likely implication of this is the stronger relation of soil loss to topography rather than to vegetation cover.

**Table 6:** Soil losses and their areal extent for the year 2000.

Soil Loss Range (tons/ha/year)	Soil Loss Class	Area (ha)	Area (%)	Soil Loss (tons/year)	Soil Loss (%)	Average Soil Loss (tons/ha/year)
0–1	Very low	16036.45	89.48	2459.65	17.27	0.15
1–5	Low	1308.49	7.30	3291.31	23.11	2.52
5–10	Moderate	333.81	1.86	2917.14	20.48	8.74
10–15	High	137.74	0.77	2083.51	14.63	15.13
15–20	Very high	47.80	0.27	1011.19	7.10	21.15
>20	Severe	56.71	0.32	2481.51	17.42	43.75
Total		17,921.00	100	14,244.31	100	91.44

**Table 7:** Soil losses and their areal extent for the year 2017.

Soil Loss Range (tons/ha/year)	Soil Loss Class	Area (ha)	Area (%)	Soil Loss (tons/year)	Soil Loss (%)	Average Soil Loss (tons/ha/year)
0–1	Very low	15519.07	86.60	2954.76	17.60	0.19
1–5	Low	1780.72	9.94	4011.94	23.89	2.25
5–10	Moderate	339.88	1.90	2934.40	17.47	8.63
10–15	High	140.67	0.78	2113.17	12.58	15.02
15–20	Very high	60.98	0.34	1304.70	7.77	21.39
>20	Severe	79.69	0.44	3473.36	20.68	43.59
Total		17,921.00	100.00	16,792.33	100.00	

Table 6, Table 7 show the soil loss and its areal extent in 2000 and 2017 respectively. More than 89 % and 86 % of the area experienced very low erosion risk in 2000 and 2017 respectively. It is observed that the annual soil loss increases in risk level from the year 2000–2017. This is demonstrated in erosion hotspot maps, where most of the areas initially identified as high erosion risk areas have turned into extreme erosion risk areas in 2017.

### 3.7 Sediment delivery into Gubi reservoir

#### 3.7.1 Sediment delivery ratio (*SDR*) and sediment yield

**Fig. 10:**
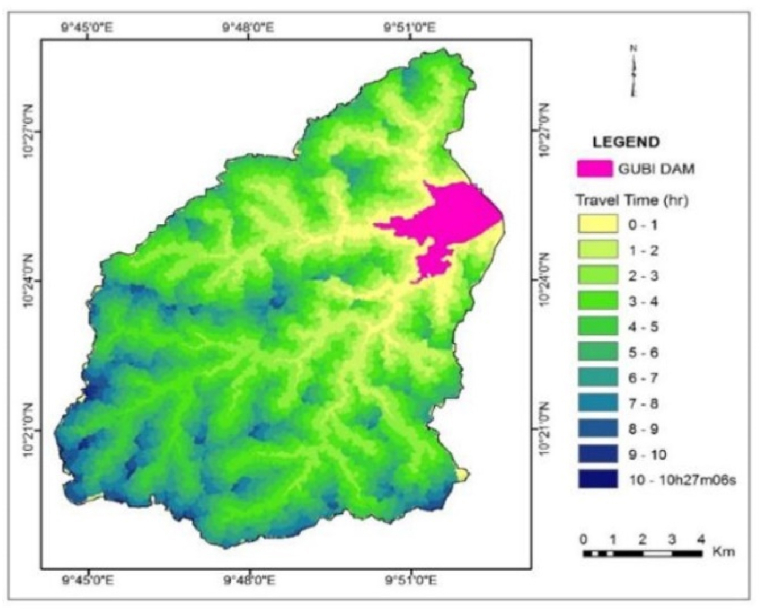
Raster map of travel time (hr).

**Fig. 11:**
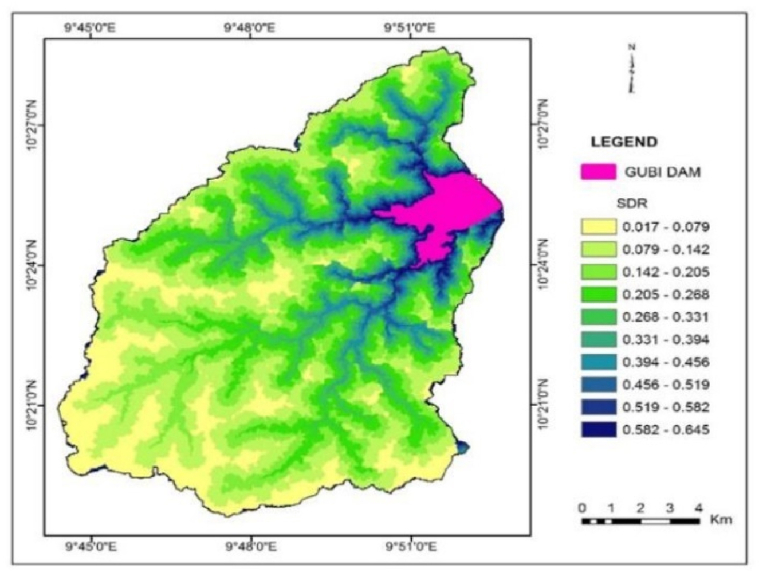
Raster map of SDR.

The result of sediment travel time shows that the time of concentration of the catchment is 10h 27m 6s (Fig. 10). As expected, little sediments from the farthest points of the watershed reach the reservoir site as indicated by low SDR values (Fig. 11) and large sediment travel times. Basins with large drainage areas and long travel times have a low sediment delivery ratio. The large area provides chances to trap soil particles thus reducing the chance of soil particles reaching the reservoir.

**Fig. 12:**
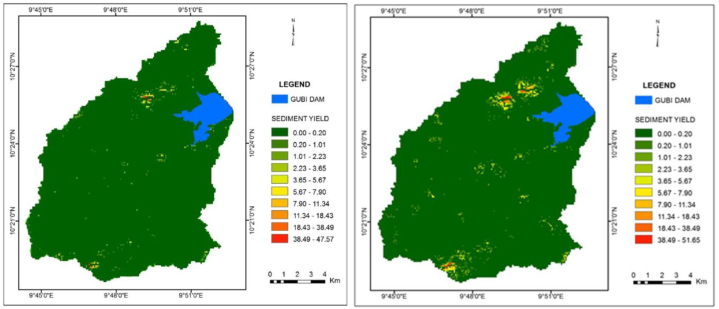
a: Sediment yield in the year 2000 Fig. 12. b: Sediment yield in the year 2017.

The total sediment yield from the Gubi watershed was 6903.65 tons/yr in 2000 and increased to 9172.71 tons/yr in 2017. They were obtained by summing up the sediment harvest of *cell*_*i-n*_ in GIS. This corresponds to the catchment's SDR of 48.46 % and 54.62 % in 2000 and 2017 respectively. The sediment yield ranges from 0 to 47.57 tons/ha/yr and 0–51.65 tons/ha/yr in 2000 and 2017 respectively (Fig. 12a and b). The highest sediment yield values were concentrated in the southern part extending as far as the central and the north tips along the eastern escarpments and these areas are the ones with the steepest slopes covered with rocks.

#### 3.7.2 Model validation

Data on sediment yield from six hydrological stations in Northern Nigeria (Table 8) was used to validate the RUSLE results. It is observed that the mean sediment yield from the six catchments was 33.46 tons/ha/yr. Even though the six catchments have larger areas compared to Gubi, the range of sediment yield compares well with the sediment yield from the Gubi catchment whose values ranged from 0 to 47.57 tons/ha/year for 2000 and 0–51.65 tons/ha/yr for 2017. However, the overall mean sediment yield from this study is low compared to those reported in Table 8.

**Table 8:** Measured sediment yields from catchments in Northern Nigeria (Source: [58]).

Hydrological Station	River	Catchment Area (ha)	Mean Annual Rainfall (mm)	Measured Sediment Yield (tons/ha/yr)
Zurmi	Bunsuru	683,200	762–1016	13.25
Kaura Namoda	Gagere	617,800	762–1016	18.37
Gusau	Sokoto	265,600	762–1143	20.91
Anka	Zamfara	413,000	762–1016	28.47
Railway Bridge	Challawa	679,400	762–1143	105.12
Chiromawa	Kano	390,800	889–1270	14.65
				Mean = 33.46

Furthermore, the results of model validation using the Fournier method gave an annual sediment yield of 42.66 tons/ha/yr which is also comparable.

#### 3.7.3 Sediment inflow into Gubi reservoir

**Fig. 13:**
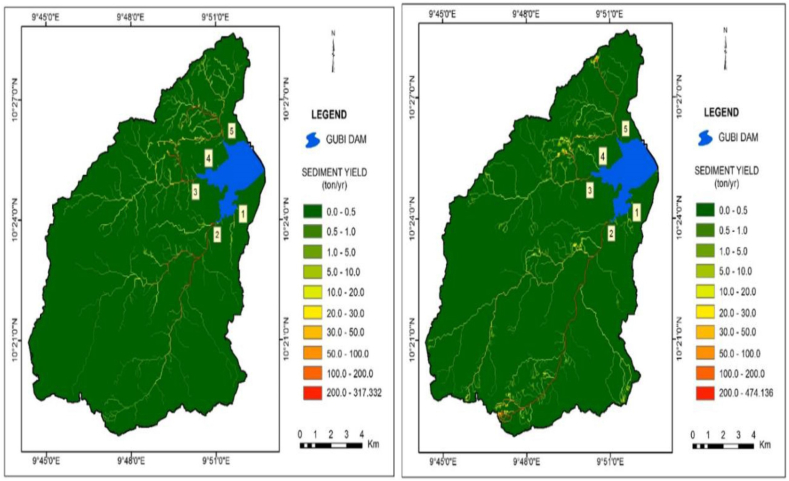
a: Sediment inflow into Gubi dam in 2000 Fig. 13. b: Sediment inflow into Gubi dam in 2017.

It is interesting to observe significant variations in sediment inflow between the two study periods. The total sediment inflow into the Gubi reservoir was 747.38 and 950.76 tons/year which is equivalent to 10.83 % and 10.37 % of the total sediment produced in the years 2000 and 2017 respectively (Fig. 13a and b). It is noted that only a small fraction of the total sediments generated from the watershed reach the reservoir as some sediments are trapped in vegetated areas, some are trapped in floodplain and some are deposited in channels.

There were significant variations in sediment inflow between the five sub-basins in the watershed. The sediment yield from all the sub-basins shows an increasing trend from 2000 to 2017. The sediment inflow from the five inlets ranged from 16.65 to 317.33 tons/year in the years 2000 and 18.61–474.14 tons/year in 2017, with Sub-basin 1 giving the least sediment and Sub-basin 2 the highest in both cases (Table 9). The land use of Gubi sub-basin has changed substantially over this time leading to major soil erosion problems.

**Table 9:** Sediment inflow into Gubi reservoir from various inlets.

Inlet	Sediment (tons) in 2000	Area (%)	Sediment (tons) in 2017	Area (%)
1	16.65	2.23	18.61	1.96
2	317.33	42.46	474.14	49.87
3	257.33	34.43	281.49	29.61
4	18.14	2.43	22.14	2.33
5	137.93	18.46	154.38	16.24
Total	747.38	100.00	950.76	100.00

## 4 Discussion

### 4.1 Rainfall erosivity factor (*R*)

In Northern Nigeria, the rainfall exhibited a progressive increase from low values at the onset of the rainy season, peaking around August, and thereafter declining to lower values towards the end of the season. July, August, and September contributed almost 80 % of annual rainstorms. Rainfall occurs during the incursion of the tropical easterlies into the study area. The easterlies, characterized by thick low-level clouds produce thunderstorms of high intensities with short duration, which have tendencies of tearing apart and transporting soil material largely by flowing water. The results of the R-factor indicated that the southern parts of the catchment have a rainfall distribution with an average R-factor ranging from 461.41 to 469.68, compared with the northern part, with a value of 428.34–436.61. The total erosive power in the southern part of the watershed is higher compared with the northern part. Furthermore, the calculated R-factor values were compared with another work by Safiyyah [59] which estimated the R-factor based on the mean annual precipitation of a state nearby (Kano) for thirty years. Since Kano receives a lesser amount of rainfall compared to the Gubi catchment, an R-factor of 143.07 MJ mm/ha/hr year was obtained which can be considered to have compared well with the current study. A study by Reshma and Uday [60] in Upper South Koel Basin, Jharkhand, India, reported an R-factor of 546 MJ mm/ha/hr/yr which also compared well with the current study.

### 4.2 Soil erodibility factor (*K*)

In general, the *K* values in the basin were low. From the comparison, the erodibility values under the very low class covers almost 121.856 km^2^ (68 % of the total land mass), and low classes carried 57.356 km^2^ (32.02 %). The main reason for the low soil erodibility lies in the soil properties such as the high presence of coarse grain size particles which reduces the soil loss because greater force is required to entrain them. On the other hand, the finer particles are resistant to detachment, because of their cohesive character, which provides greater resistance to reduce erosion. The finding also is in agreement with the study done by Safiyyah [59] in Kano (Nigeria), which reported that the soil erodibility factor was 0.4127, 0.7581, 0.3288, and 0.3968 in four irrigation sectors. The K-factors were within the range of 0.02–0.69 as given by Goldman and Jackson [61] except for one area. Adediji et al. [62] also reported K-values ranging from 0.04 to 0.13 in Katsina (Nigeria). Rashma and Uday [60] also reported a soil erodibility K-factor varying from 0.23 to 0.37 in Jharkhand (India).

### 4.3 Slope length and slope steepness factor (*LS*)

The slope of the Gubi watershed has high *LS* variability indicating the differences in their effect on soil loss. Most of the extreme southwest, central, and northeastern parts were observed to have high values with their eminent contribution towards soil loss, but the relatively flat areas have marginally higher values since such areas extend longer with uniform steepness. Soil erosion increases more with an increase in slope and slightly increases with slope length. Thus, slope wash increases with slope steepness and slope length, owing to the increase in velocity and surface runoff. This finding was supported by Adedeji et al. [62], who reported that soil loss increases more rapidly with slope steepness than it does with slope length. This is because the areas mostly affected by erosion within the study area coincided with the areas where the LS-factor is high. Kouli et al. [21] used the RUSLE model and their results show that the topographic factor ranges from 0 in flatter zones to 118 at the steeper slopes.

### 4.4 Vegetation cover factor and support practice (*C*)

The C-factor, derived from the vegetation indices, has shown strong relationships with the land use of the watershed. There is a wide variation of the *C* values with their non-uniformity in spatial distribution within the two periods. During 2000, the study area experienced a high vegetation cover, corresponding to low *C* values, which are found mostly along the southern valley through which the river flows, similarly, the steep lands of the eastern escarpments, and along watercourses. The most likely reason for the high vegetation cover along the valley and watercourses is the retention of moisture for comparatively longer periods. Subsequently, during the same year, low vegetation covers were observed, corresponding to high *C* values, which were seen predominantly in the central and southern parts of the watershed. Furthermore, it is observed that the study area was almost covered with more severe to exceptional erosion in 2017 compared to 2000. This shows that the land use of the watershed has changed substantially over the time frame, leading to major soil erosion problems. The forest area was rapidly deforested and substituted with agriculture and urban development. The forest area reduced from 32.65 % in 2000 to 21.30 % in 2017, leaving the land exposed and bare, posing a huge erosion risk. The largest contributor to soil erosion in the watershed is agricultural activities, which covered 59.75 % of the area in the year 2000 and increased to 67.66 % area in 2017. The built-up area increased from 1.91 % in 2000 to 3.19 % in 2017. This agrees with Teh [19] and Da and Jon [63] suggesting that agricultural activity is the main contributor to soil erosion followed by urbanization.

### 4.5 Average annual soil loss (*A*)

The average annual soil loss estimated for the entire watershed was 0.795 tons/ha/yr in the year 2000 and 0.937 tons/ha/yr in 2017. The soil loss was generally low when compared with Adedeji et al. [62] who estimated 6.94 tons/ha/yr for Katsina, Nigeria. However, the finding is in agreement with Safiyyah [59] in Kano (Nigeria), who reported an estimated soil loss of 1.62, 3.47, 1.39, and 1.71 tons/hectare/year for four irrigation sectors in Kano. Similarly, this finding was supported by a similar study in Upper South Koel Basin, Jharkhand (India) [60], where an annual soil loss of 12.2 tons/ha/yr was estimated using the USLE. It was emphasized that the agricultural lands with an increase in slope gradient experience more soil loss. The spatial distribution of soil loss between the two study periods shows that most of the steep areas of the southwestern part, central and the northeastern escarpments have high erosion potentials. There is likely a stronger relationship between soil loss and topography than vegetation cover. Wardafo [7] also concluded that topography along with erodibility had the highest effect on soil loss calculated with the RUSLE. Similarly, Adedeji [62] noted that areas with higher steepness factors gave the highest soil loss.

### 4.6 Sediment delivery to the Gubi reservoir

The sediment yield at the Gubi basin increased from 6903.65 tons in 2000–9172.71 tons in 2017. It is noted that from the year 2000–2017, there was an increase in sediment yield by 2269.06 tons which is equivalent to 32.87 %. This is consistent with the finding of Lindsay [64] which reported the erosion sources within the Emory & Henry College Duck Pond Watershed and predicted the annual sediment yield of 1076 tons that was expected to accumulate at the pond. The RUSLE model predicted an average soil loss of approximately twice the sediment yield because some sediments are trapped in vegetated areas, some are trapped in floodplain and some are deposited in channels, but some portion of eroded material is delivered to the outlet of the watershed. Results show that all of the sub-watersheds of the Gubi River have an increasing trend in sediment yield from 2000 to 2017. Among the sub-watershed, Inlet 2 (Fig. 13a and b) has the highest predicted average annual sediment delivered to the reservoir in both the two periods. This is due to its large drainage area conveying more soil particles to the water channel system. The sediment inflow from this inlet that reached the reservoir in the year 2000 was 317.33 tons/year, which increased to 474.14 tons/year in 2017 constituting an increase by about half of the total sediment delivery of year 2000.

Unsustainable practices such as forest clearing for firewood have resulted in extensive deforestation leading to sparse vegetation and poor forest cover. The people also engage in agricultural cultivation, overgrazing, and rapid infrastructural development within the catchments, which increases the soil loss. Da and Jon [65] reported that LULC is another factor affecting SDR. A watershed with good vegetation cover has a low SDR because vegetation slows down the runoff rate and allows the eroded soil practices to deposit. Teh [19] also reported a similar finding in Malaysia, that the development of sediment yield is very closely related to the change in land use, the agricultural management situation, along with terrain features such as slope length and slope steepness.

The sediment deposition rate in the Gubi reservoir in the year 2000 was estimated to be 747.38 tons/yr, which is equivalent to 10.83 % of the total sediment produced that year. The total deposited sediment in 2017 was 950.76 tons/year, which corresponds to 10.37 % when compared with the total generated sediment. Even though the Gubi reservoir is not used for hydroelectric power generation, sediment deposition could affect its potential to be used for hydroelectric power. The annual rate of sediment inflow into the Gubi reservoir has increased, which consequently affects its storage capacity, thus reducing its useful life. Therefore, major erosion contributors must be tackled to solve capacity loss problems at the Gubi reservoir. Furthermore, the results of this study can be used to develop soil and water conservation measures.

### 4.7 Limitations and model validation

Model validation involves comparing the results from the simulation to real-life measured soil loss data. However, real-life soil loss data from catchments is difficult to collect as ground-truthing a large watershed and collecting soil loss data under different conditions is necessary. Thus, the validation of RUSLE results constitutes one of the limitations of its use. Furthermore, the catchment is located in sub-Saharan Africa, where data is scarce. Many researchers who used RUSLE have pointed out this issue. Olika et al. [13] and Mathewos et al. [66] indicated the problem of a lack of readily available data on soil loss which makes the validation of RUSLE results difficult. For these reasons, many researchers have used RUSLE for soil loss modeling without validating their results [[67], [68], [69]].

## 5 Conclusion

In this research, RUSLE was used to estimate the soil loss over the 17,921 ha Gubi catchment and to determine the sediment delivery into the Gubi reservoir. The results indicate that more than 89 % and 86 % of the area experienced very low erosion risks in 2000 and 2017 respectively. However, the erosion risk is expected to increase with time, since the forested area is rapidly being deforested and converted to agricultural and urban areas. There was an average soil loss of 14,244.32 tons/year in 2000 and 16,792.33 tons/year in 2017. The total sediment inflow into Gubi reservoir in 2000 was estimated to be 747.38 tons/year which is equivalent to 10.83 % of the total sediment produced. Similarly, the total sediment inflow in 2017 was 950.76 tons/year which corresponds to 10.37 % of the total sediment yield in that year. The sediment delivery is expected to increase with time as deforestation, agricultural activities, and urbanization increase. Soil erosion control measures, such as tree planting, mulching, contour ridging, and strip cropping, are recommended for soil conservation. It is recommended that a bathymetric survey be carried out to determine the extent of reservoir sedimentation to determine the amount of sediment that has been deposited to guide the reservoir operators during maintenance activities such as dredging, etc.

## Data availability

Data used in carrying out this work is available in the Supplementary File.

## Ethics declaration

Not applicable.

## CRediT authorship contribution statement

**Ali Aldrees:** Writing – review & editing, Writing – original draft, Validation, Funding acquisition, Formal analysis, Data curation. **Samaila Jibrin El-pateh:** Writing – original draft, Visualization, Validation, Software, Methodology, Conceptualization. **Salisu Dan'azumi:** Writing – review & editing, Supervision, Project administration, Methodology, Investigation. **Sani Isah Abba:** Visualization, Validation, Software, Methodology, Formal analysis, Data curation.

## Declaration of competing interest

The authors declare that they have no known competing financial interests or personal relationships that could have appeared to influence the work reported in this paper.
