# Supplementary material for: Spatio-temporal soil loss modelling using RUSLE and sediment delivery into a reservoir in a semi-arid region of northern Nigeria
Source: Heliyon. 2024 Oct 5;10(20):e38887. doi: 10.1016/j.heliyon.2024.e38887 (PMC11530838; doi:10.1016/j.heliyon.2024.e38887)
Supplement: Multimedia component 1 [file mmc1.docx]

| **SUPPLEMENTARY FILE 1**  **Tables S1a to S1e (Data for Rainfall Erosivity)** | | | | | | | | | | | | | |
| --- | --- | --- | --- | --- | --- | --- | --- | --- | --- | --- | --- | --- | --- |
| Tabla S1a: Monthly Rainfall Data (mm) Obtained from Bauchi State Agricultural Development Project, zonal office,  Bauchi (1995-2017) | | | | | | | | | | | | | |
| Year | Jan | Feb | Mar | Apr | May | Jun | Jul | Aug | Sep | Oct | Nov | Dec | Annual Rainfall |
| 1995 | 0 | 0 | 7.5 | 59.4 | 123.4 | 120.9 | 235.2 | 262.4 | 194.2 | 18.3 | 0 | 0 | 1,021.30 |
| 1996 | 0 | 0.4 | 0 | 32.4 | 93.6 | 143.4 | 175.4 | 343.9 | 263.9 | 21.9 | 0 | 0 | 1,074.90 |
| 1998 | 0 | 0 | 0 | 15.7 | 64.3 | 248.3 | 272.9 | 330.7 | 145.1 | 71.8 | 0 | 0 | 1,148.80 |
| 1999 | 0 | 0 | 0 | 7.9 | 82.5 | 159.9 | 352.1 | 333.9 | 287.6 | 48.7 | 0 | 0 | 1,272.60 |
| 2000 | 0 | 0 | 7.7 | 74.2 | 162.1 | 185.2 | 226 | 301.6 | 159.7 | 19.8 | 6.4 | 0 | 1,142.70 |
| 2001 | 0 | 0 | 0 | 15.8 | 64.3 | 148.3 | 372.9 | 330.7 | 245.1 | 71.8 | 0 | 0 | 1,248.90 |
| 2002 | 0 | 0 | 28 | 9.5 | 107.8 | 178.6 | 243.7 | 222.1 | 215.6 | 36.5 | 0 | 0 | 1,041.80 |
| 2003 | 0 | 0 | 0 | 21.6 | 74.8 | 212.6 | 299.3 | 367.2 | 133.3 | 48.8 | 0 | 0 | 1,157.60 |
| 2004 | 0 | 0 | 1.5 | 31.6 | 89.8 | 277.7 | 265.4 | 238.3 | 347 | 4.3 | 0 | 0 | 1,255.60 |
| 2005 | 0 | 0 | 0 | 25 | 107 | 184 | 81 | 350 | 161 | 31.1 | 0 | 0 | 939.10 |
| 2006 | 0 | 0.5 | 0 | 0 | 170 | 192 | 217.5 | 274 | 143 | 35.5 | 0 | 0 | 1,032.50 |
| 2007 | 0 | 0 | 0 | 48 | 0 | 111 | 294 | 483 | 158 | 4 | 0 | 0 | 1,098.00 |
| 2008 | 0 | 0 | 0 | 6 | 82 | 188.5 | 182 | 250 | 58 | 66 | 0 | 0 | 832.50 |
| 2009 | 0 | 0 | 0 | 120 | 103 | 160 | 152.2 | 240.1 | 310.5 | 145.7 | 0 | 0 | 1,231.50 |
| 2010 | 0 | 0 | 0.5 | 16 | 53.3 | 152.1 | 208.7 | 239.7 | 303.2 | 100.8 | 0 | 0 | 1,074.30 |
| 2011 | 0 | 0 | 0 | 21.5 | 55 | 158.4 | 211 | 269.4 | 221.3 | 59.6 | 0 | 0 | 996.20 |
| 2012 | 0 | 0 | 0 | 24.2 | 181.3 | 87.5 | 318.8 | 298.8 | 289 | 27 | 0 | 0 | 1,226.60 |
| 2013 | 0 | 0 | 0 | 27.5 | 62.8 | 189.5 | 434 | 494 | 153 | 79 | 0 | 0 | 1,439.80 |
| 2014 | 0 | 2 | 2 | 19.1 | 112.1 | 157.8 | 181.8 | 179 | 310.6 | 41.5 | 0 | 0 | 1,005.90 |
| 2015 | 0 | 0 | 0 | 67 | 93 | 148 | 134.5 | 210.6 | 205.7 | 44 | 0 | 0 | 902.80 |
| 2016 | 0 | 0 | 3 | 24.3 | 73 | 183.8 | 201.6 | 253.2 | 211.7 | 120 | 0 | 0 | 1,070.60 |
| 2017 | 0 | 0 | 0 | 31.2 | 46 | 123 | 217.3 | 348 | 132.1 | 8 | 0 | 0 | 905.60 |
| **AVERAGE ANNUAL RAINFALL (mm)** | | | | | | | | | | | | | **1,096.35** |
|  |  |  |  |  |  |  |  |  |  |  |  |  |  |

| Table S1b: Monthly Rainfall Data Obtained from Bauchi Old Airport Station, Nigerian Metrological Agency (NIMET) (1990 – 2017) | | | | | | | | | | | | | |
| --- | --- | --- | --- | --- | --- | --- | --- | --- | --- | --- | --- | --- | --- |
| Year | Jan | Feb | March | April | May | Jun | Jul | Aug | Sep | Oct | Nov | Dec | Annual Rainfall |
| 1990 | 0 | 0 | 0 | 5.9 | 108.7 | 100.5 | 284.7 | 262.3 | 87.8 | 29.7 | 0 | 0 | 879.60 |
| 1991 | 0 | 0 | 28.5 | 85.9 | 149.1 | 103.3 | 283.1 | 244.7 | 35.9 | 19.1 | 0 | 0 | 949.60 |
| 1992 | 0 | 0 | 2.4 | 49.4 | 50.2 | 177.5 | 328 | 357.6 | 233.5 | 28.1 | 2.7 | 0 | 1,229.40 |
| 1993 | 0 | 0 | 0 | 14.9 | 81.3 | 236.1 | 231.8 | 337.7 | 179.5 | 60.6 | 0 | 0 | 1,141.90 |
| 1994 | 0 | 0 | 0 | 106.9 | 58.4 | 56.4 | 225.2 | 387.4 | 295.7 | 44.2 | 0 | 0 | 1,174.20 |
| 1995 | 0 | 0 | 0 | 27.2 | 104.8 | 197 | 123.1 | 277.1 | 212.8 | 19.4 | 0 | 0 | 961.40 |
| 1996 | 0 | 0 | 0 | 18.1 | 41 | 151.2 | 237.2 | 341.4 | 261.8 | 44.6 | 0 | 0 | 1,095.30 |
| 1997 | 0 | 0 | 0 | 27.3 | 95 | 182.3 | 249.2 | 231.6 | 182.2 | 16.6 | 0 | 0 | 984.20 |
| 1998 | 0 | 0 | 0 | 11.5 | 184.6 | 139.8 | 308.1 | 329.3 | 254.7 | 43.81 | 0 | 0 | 1,271.81 |
| 1999 | 0 | 0 | 0 | 2.7 | 41.2 | 97.9 | 440 | 344 | 262.4 | 186.3 | 0 | 0 | 1,374.50 |
| 2000 | 0 | 0 | 0 | 10.3 | 80.5 | 142.1 | 251.6 | 308.5 | 125.2 | 20.9 | 0 | 0 | 939.10 |
| 2001 | 0 | 0 | 0 | 1.2 | 155.3 | 274.4 | 324.8 | 354.1 | 199.9 | 37.2 | 0 | 0 | 1,346.90 |
| 2002 | 0 | 0 | 0 | 76.9 | 26.6 | 112.2 | 155.4 | 238.9 | 287.4 | 53.8 | 0 | 0 | 951.20 |
| 2003 | 0 | 0 | 0 | 31 | 93 | 295 | 124.4 | 263.5 | 173.9 | 29.7 | 0 | 0 | 1,010.50 |
| 2004 | 0 | 0 | 1.9 | 33.8 | 87.8 | 277.1 | 267.4 | 138.3 | 449 | 41 | 0 | 0 | 1,296.30 |
| 2005 | 0 | 0 | 0 | 24.8 | 100.8 | 240.2 | 157.9 | 364 | 166.5 | 24.3 | 0 | 0 | 1,078.50 |
| 2006 | 0 | 0 | 0 | 0 | 157.5 | 155.9 | 217.3 | 229.5 | 146.5 | 36 | 0 | 0 | 942.70 |
| 2007 | 0 | 0 | 0 | 27.6 | 17.3 | 121.3 | 280.5 | 520.9 | 162.3 | 7 | 0 | 0 | 1,136.90 |
| 2008 | 0 | 0 | 0 | 9.2 | 77.8 | 337.1 | 216.5 | 394.7 | 57.1 | 42.7 | 0 | 0 | 1,135.10 |
| 2009 | 0 | 0 | 0 | 162.6 | 106.2 | 184.8 | 211.6 | 403.8 | 288.9 | 173.4 | 0 | 0 | 1,531.30 |
| 2010 | 0 | 0 | 0 | 41.9 | 74.8 | 203.2 | 446 | 219 | 489.3 | 146.9 | 0 | 0 | 1,621.10 |
| 2011 | 0 | 0 | 0 | 60.3 | 76.8 | 139.7 | 210.8 | 624.7 | 333.7 | 110.8 | 0 | 0 | 1,556.80 |
| 2012 | 0 | 0 | 0 | 14.4 | 167.9 | 126.4 | 535.3 | 302 | 375.3 | 16.4 | 0 | 0 | 1,537.70 |
| 2013 | 0 | 0 | 0 | 41.7 | 61.9 | 295 | 569 | 705.8 | 126 | 114.7 | 0 | 0 | 1,914.10 |
| 2014 | 0 | 16.8 | 1.6 | 85.3 | 170.4 | 253 | 347 | 406.5 | 218.8 | 15.2 | 0 | 0 | 1,514.60 |
| 2015 | 0 | 0 | 5.2 | 11.6 | 123.2 | 139.1 | 173.8 | 198.2 | 238.2 | 61.4 | 0 | 0 | 950.70 |
| 2016 | 0 | 0 | 32.8 | 20.8 | 139.2 | 173 | 236.7 | 257.9 | 163.5 | 26.1 | 0 | 0 | 1,050.00 |
| 2017 | 0 | 0 | 2.3 | 19.5 | 46.3 | 176.1 | 198.2 | 355.1 | 143.9 | 13.4 | 0 | 0 | 954.80 |
| **AVERAGE ANNUAL RAINFALL (mm)** | | | | | | | | | | | | | **1,241.86** |
|  |  |  |  |  |  |  |  |  |  |  |  |  |  |
|  |  |  |  |  |  |  |  |  |  |  |  |  |  |
| Table S1c: Monthly Rainfall Data Obtained from College of Agriculture, Bauchi (1998 – 2017) | | | | | | | | | | | | | |
| Year | Jan | Feb | Mar | Apr | May | Jun | Jul | Aug | Sep | Oct | Nov | Dec | Annual Rainfall |
| 1998 | 0 | 0 | 0.7 | 23.4 | 132.6 | 98.2 | 163.2 | 301.9 | 285.5 | 27.7 | 0 | 0 | 1,033.20 |
| 1999 | 0 | 0 | 1.3 | 17.3 | 121.6 | 97.6 | 225.1 | 425 | 211.6 | 23.6 | 0 | 0 | 1,123.10 |
| 2000 | 0 | 0 | 0.8 | 56.6 | 141.7 | 168 | 249.3 | 334.7 | 123.1 | 16.8 | 0 | 0 | 1,091.00 |
| 2001 | 0 | 0 | 0 | 31.8 | 34.4 | 120.2 | 213.7 | 370 | 204 | 41.3 | 0 | 0 | 1,015.40 |
| 2002 | 0 | 0 | 11.5 | 17.6 | 131.1 | 171.8 | 318.7 | 310.2 | 185.8 | 38.1 | 0 | 0 | 1,184.80 |
| 2003 | 0 | 0 | 0 | 13.7 | 77.3 | 114.4 | 354.5 | 306.3 | 224.1 | 50.2 | 0 | 0 | 1,140.50 |
| 2004 | 0 | 0 | 28.3 | 31.6 | 73.3 | 106 | 226 | 363.4 | 230.7 | 45.7 | 0 | 0 | 1,105.00 |
| 2005 | 0 | 0 | 0 | 27.3 | 142.3 | 181.2 | 124 3 | 330.4 | 117.4 | 16.4 | 0 | 0 | 815.00 |
| 2006 | 0 | 0 | 0 | 20.6 | 74.8 | 212.8 | 299.6 | 367.2 | 133.1 | 42.8 | 0 | 0 | 1,150.90 |
| 2007 | 0 | 0 | 0 | 37.2 | 75.1 | 83.5 | 329.4 | 258.7 | 165.7 | 50.7 | 0 | 0 | 1,000.30 |
| 2008 | 0 | 0 | 0 | 3.6 | 111.6 | 121.3 | 214.3 | 317.9 | 82.5 | 40.3 | 0 | 0 | 891.50 |
| 2009 | 0 | 0 | 0 | 76.6 | 120.2 | 206.3 | 296.3 | 348.5 | 292.3 | 48.1 | 0 | 0 | 1,388.30 |
| 2010 | 0 | 0 | 5.6 | 43.8 | 38.4 | 164.9 | 233.2 | 239.2 | 241 | 31.6 | 0 | 0 | 997.70 |
| 2011 | 0 | 0 | 0 | 34.2 | 95.4 | 123.5 | 227.1 | 284.2 | 176.3 | 75,2 | 0 | 0 | 940.70 |
| 2012 | 0 | 0 | 6.8 | 19.3 | 148.7 | 77.8 | 339.8 | 227.5 | 264.1 | 10.3 | 0 | 0 | 1,094.30 |
| 2013 | 0 | 0 | 0 | 33.2 | 102.3 | 148.4 | 352.2 | 382.6 | 145.6 | 83.2 | 0 | 0 | 1,247.50 |
| 2014 | 0 | 0 | 5.7 | 8 | 86.1 | 129.5 | 242.8 | 171.1 | 228.9 | 20.3 | 0 | 0 | 892.40 |
| 2015 | 0 | 0 | 0 | 56.3 | 107.7 | 219.3 | 151.1 | 240.4 | 231.2 | 46.3 | 0 | 0 | 1,052.30 |
| 2016 | 0 | 0.5 | 1.8 | 66.6 | 36.8 | 132.6 | 298 | 289.1 | 129.9 | 33.8 | 0 | 0 | 989.10 |
| 2017 | 0 | 0 | 0 | 26.6 | 99.7 | 177.6 | 182.3 | 324.7 | 140.8 | 57.4 | 0 | 0 | 1,009.10 |
| **AVERAGE ANNUAL RAINFALL (mm)** | | | | | | | | | | | | | **1,113.79** |
|  |  |  |  |  |  |  |  |  |  |  |  |  |  |
|  |  |  |  |  |  |  |  |  |  |  |  |  |  |

| Table S1d: Monthly Rainfall Data Obtained from School General Studies, Abubakar Tatari Ali Polytechnic, Bauchi, (2009 – 2016) | | | | | | | | | | | | | |
| --- | --- | --- | --- | --- | --- | --- | --- | --- | --- | --- | --- | --- | --- |
| Year | Jan | Feb | Mar | Apr | May | Jun | Jul | Aug | Sep | Oct | Nov | Dec | Annual Rainfall |
| 2009 | 0 | 0 | 0 | 83.3 | 118.5 | 157.7 | 205.1 | 255.6 | 240.8 | 63.3 | 0 | 0 | 1,124.30 |
| 2010 | 0 | 0 | 0 | 40 | 39.9 | 119.3 | 216.9 | 186.7 | 253.1 | 29.2 | 0 | 0 | 885.10 |
| 2011 | 0 | 0 | 5.1 | 32.8 | 118.5 | 129.7 | 241.4 | 272.5 | 237.6 | 73,1 | 0 | 0 | 1,037.60 |
| 2012 | 0 | 0 | 0 | 12.7 | 151.2 | 172.9 | 286.3 | 236.7 | 197.1 | 30.4 | 0 | 0 | 1,087.30 |
| 2013 | 0 | 0 | 0 | 48.5 | 97.6 | 169.2 | 312.6 | 349.9 | 222.2 | 32.2 | 0 | 0 | 1,232.20 |
| 2014 | 0 | 0.9 | 5.1 | 20.5 | 97.6 | 155.2 | 235.4 | 335.5 | 251.4 | 35.3 | 0 | 0 | 1,136.90 |
| 2015 | 0 | 0 | 2.4 | 38.9 | 51.2 | 186.3 | 236.3 | 217.6 | 174.5 | 21.3 | 0 | 0 | 928.50 |
| 2016 | 0 | 0 | 9.7 | 22.1 | 67.4 | 223.6 | 239.9 | 272.8 | 251.1 | 84.5 | 0 | 0 | 1,171.10 |
| **AVERAGE ANNUAL RAINFALL (mm)** | | | | | | | | | | | | | **1,229.00** |
|  |  |  |  |  |  |  |  |  |  |  |  |  |  |
|  |  |  |  |  |  |  |  |  |  |  |  |  |  |
|  |  |  |  |  |  |  |  |  |  |  |  |  |  |
| Table S1e: Monthly Rainfall Data Obtained from Nigerian Metrological Agency (NIMET), New Airport, Bauchi, (2011 – 2017) | | | | | | | | | | | | | |
| Year | Jan | Feb | Mar | Apr | May | Jun | Jul | Aug | Sep | Oct | Nov | Dec | Annual Rainfall |
| 2011 | 0 | 0 | 2.5 | 40.3 | 127.5 | 108.6 | 268.2 | 293.1 | 249.2 | 59.5 | 0 | 0 | 1,148.90 |
| 2012 | 0 | 0 | 0 | 22.7 | 145,2 | 178.2 | 264.8 | 263 | 285.4 | 45.7 | 0 | 0 | 1,059.80 |
| 2013 | 0 | 0 | 0 | 54.1 | 108.3 | 178.3 | 336.1 | 334.8 | 239.3 | 54.1 | 0 | 0 | 1,305.00 |
| 2014 | 0 | 1.7 | 7.5 | 18.3 | 85.9 | 174.2 | 245.6 | 320.1 | 263.7 | 55,1 | 0 | 0 | 1,117.00 |
| 2015 | 0 | 0 | 1.5 | 42.6 | 64.6 | 163.6 | 257.8 | 198.3 | 187.7 | 36.1 | 0 | 0 | 952.20 |
| 2016 | 0 | 0 | 11.2 | 21.3 | 58.7 | 241.5 | 214.9 | 257.3 | 271.4 | 78.9 | 0 | 0 | 1,155.20 |
| 2017 | 0 | 0 | 0 | 35.6 | 45.9 | 189.4 | 336.8 | 327.1 | 271.8 | 11.7 | 0 | 0 | 1,218.30 |
| **AVERAGE ANNUAL RAINFALL (mm)** | | | | | | | | | | | | | **1,136.63** |

**SUPPLEMENTARY FILE 2**

**TABLES S2a to S2d (Data for soil Erodibility)**

Table S2a: Percentage gravel, sand, silt & clay for Gubi watershed

| LOCATION | | GRAVEL | SAND | | |  | |
| --- | --- | --- | --- | --- | --- | --- | --- |
| Sample no. | Sample label | Fine Gravel (%) | Coarse Sand (%) | Medium Sand  (%) | Fine Sand (%) | Silt   (%) | Clay  (%) |
| 1 | Wuntin Dada | 24 | 33 | 35 | 6 | 2 | 0 |
| 2 | Zamfara | 16 | 29 | 32 | 20 | 3 | 0 |
| 3 | Rehu 1 | 11 | 26 | 40 | 21 | 3 | 0 |
| 4 | Rehu 2 | 9 | 20 | 43 | 23 | 5 | 0 |
| 5 | Lafiyari | 5 | 38 | 34 | 19 | 4 | 0 |
| 6 | Guru 2 | 8 | 36 | 35 | 19 | 2 | 0 |
| 7 | Guru 1 | 7 | 25 | 37 | 25 | 6 | 0 |
| 8 | Rafin makaranta | 19 | 34 | 28 | 15 | 4 | 0 |
| 9 | Amour barrack | 19 | 25 | 30 | 21 | 5 | 0 |
| 10 | Kundum 2 | 20 | 29 | 31 | 16 | 4 | 0 |
| 11 | Kundum 3 | 20 | 27 | 32 | 18 | 2 | 1 |
| 12 | Kundum 1 | 7 | 28 | 32 | 24 | 9 | 0 |
| 13 | Kundum 4 | 18 | 30 | 27 | 18 | 7 | 0 |
| 14 | Sabon gari | 14 | 27 | 38 | 13 | 7 | 1 |
| 15 | Gubi west | 16 | 33 | 29 | 15 | 7 | 0 |

Table S2b: Soil structure codes for each soil sample

| Sample no | Sample label | Textural Class | Soil structure code |
| --- | --- | --- | --- |
| 1 | Wuntin Dada | Silty sand | 2 |
| 2 | Zamfara | Loamy sand | 1 |
| 3 | Rehu 1 | Sand | 1 |
| 4 | Rehu 2 | Loamy sand | 1 |
| 5 | Lafiyari | Sand | 1 |
| 6 | Guru 2 | Sand | 1 |
| 7 | Guru 1 | Sand | 1 |
| 8 | Rafin makaranta | Loamy sand | 1 |
| 9 | Amour barrack | Loamy sand | 1 |
| 10 | Kundum 2 | Silty sand | 2 |
| 11 | Kundum 3 | Loamy sand | 1 |
| 12 | Kundum 1 | Loamy sand | 1 |
| 13 | Kundum 4 | Loamy sand | 1 |
| 14 | Sabon gari | Loamy sand | 1 |
| 15 | Gubi west | Loamy sand | 1 |

Table S2c: Permeability class for the soil samples

| Sample no. | Sample Label | Soil permeability class |
| --- | --- | --- |
| 1 | Wuntin Dada | 2 |
| 2 | Zamfara | 1 |
| 3 | Rehu 1 | 1 |
| 4 | Rehu 2 | 1 |
| 5 | Lafiyari | 1 |
| 6 | Guru 2 | 1 |
| 7 | Guru 1 | 1 |
| 8 | Rafin makaranta | 1 |
| 9 | Amour barrack | 1 |
| 10 | Kundum 2 | 2 |
| 11 | Kundum 3 | 1 |
| 12 | Kundum 1 | 1 |
| 13 | Kundum 4 | 1 |
| 14 | Sabon gari | 1 |
| 15 | Gubi west | 1 |

Table S2d M values for the soil samples

| Sample no. | Sample Label | M Values |
| --- | --- | --- |
| 1 | Wuntin Dada | 800 |
| 2 | Zamfara | 2300 |
| 3 | Rehu 1 | 2400 |
| 4 | Rehu 2 | 2800 |
| 5 | Lafiyari | 2300 |
| 6 | Guru 2 | 2100 |
| 7 | Guru 1 | 3100 |
| 8 | Rafin makaranta | 1900 |
| 9 | Amour barrack | 2600 |
| 10 | Kundum 2 | 2000 |
| 11 | Kundum 3 | 1980 |
| 12 | Kundum 1 | 3300 |
| 13 | Kundum 4 | 2500 |
| 14 | Sabon gari | 1980 |
| 15 | Gubi west | 2200 |

M***=*** (%Silt + %Fine sand) × (100-%Clay)

**SUPPLEMENTARY FILE 3**

1. **Particle Size Analysis by Simple Dry Sieving BS 1377: Part 2:1990**

The laboratory texture analysis of the soil samples was undertaken using the Particle Size Analysis by Simple Dry Sieving BS 1377: Part 2:1990 and hydrometer method. The results were assigned textural names, in turn, to be refined according to the requirements of the RUSLE guideline (Table S3a).

Table S3a: Textural assignments of the RUSLE

| Texture class | Size (mm) |
| --- | --- |
| Sand  Very fine sand  Silt  Clay | 0.1-2.0  0.05-0.1  0.002-0.05  <0.002 |

     Source: (Renard et al, 1997)

1. **Determination of Soil Structure Code**

The soil structure code was determined using Figure S3a which shows the soil textural classification triangle, (Ontario Center for Soil Resource Evaluation, 1993). Percentage clay and sand in the samples were used to determine the soil structure codes for each sample i.e. (Table S3b)

     Table S3b: Soil structure class and their values

| Soil structures | Structure Class |
| --- | --- |
| Very fine granular    Fine granular  Medium or coarse granular  Blocky, platy or massive | 1  2  3  4 |

Source :(Ontario Center for Soil Resource Evaluation,1993)

***
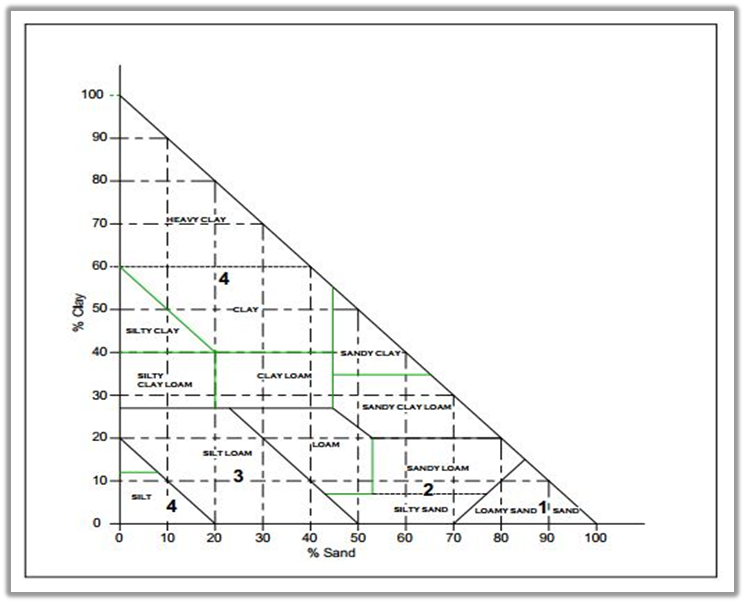
***

*Figure S3a: Soil textural classification triangle (Ontario Center for Soil Resource Evaluation, 1993)*

1. **Determination of Permeability Class of Each Soil Sample**

The soil texture classes were used to assign soil permeability values of the RUSLE guideline. The permeability class was determined using Figure S3b from soil textural classification triangle, (Ontario Center for Soil Resource Evaluation, 1993) which gives different soil textures and their permeability class. Table S3c shows the permeability class of different types of soils and a class was chosen based on the type of soil for each sample.

Table S3c: Soil permeability code based on textural class

| Soil Type | Permeability Code |
| --- | --- |
| Heavy clay, Clay | 6 |
| Silty clay loam, Sandy loam | 5 |
| Sandy clay loam, clay loam | 4 |
| Loam, Silt loam, Silty sand | 3 |
| Loamy sand, Sandy loam | 2 |
| Sand | 1 |

Source :(Ontario Center for Soil Resource Evaluation,1993)

where: 1 = rapid, 2 = moderate to rapid, 3 = moderate, 4 = slow to moderate, 5 = slow and 6 = very slow

These permeability values were given based on the contribution of the soil texture class towards generating surface runoff. Thus, soils with low infiltration and permeability capacity have high runoff potential and hence are given higher permeability value with maximum of 6 and soils with high infiltration and permeability capability have low surface runoff, which are given low permeability values that could reach to the minimum of 1.

*
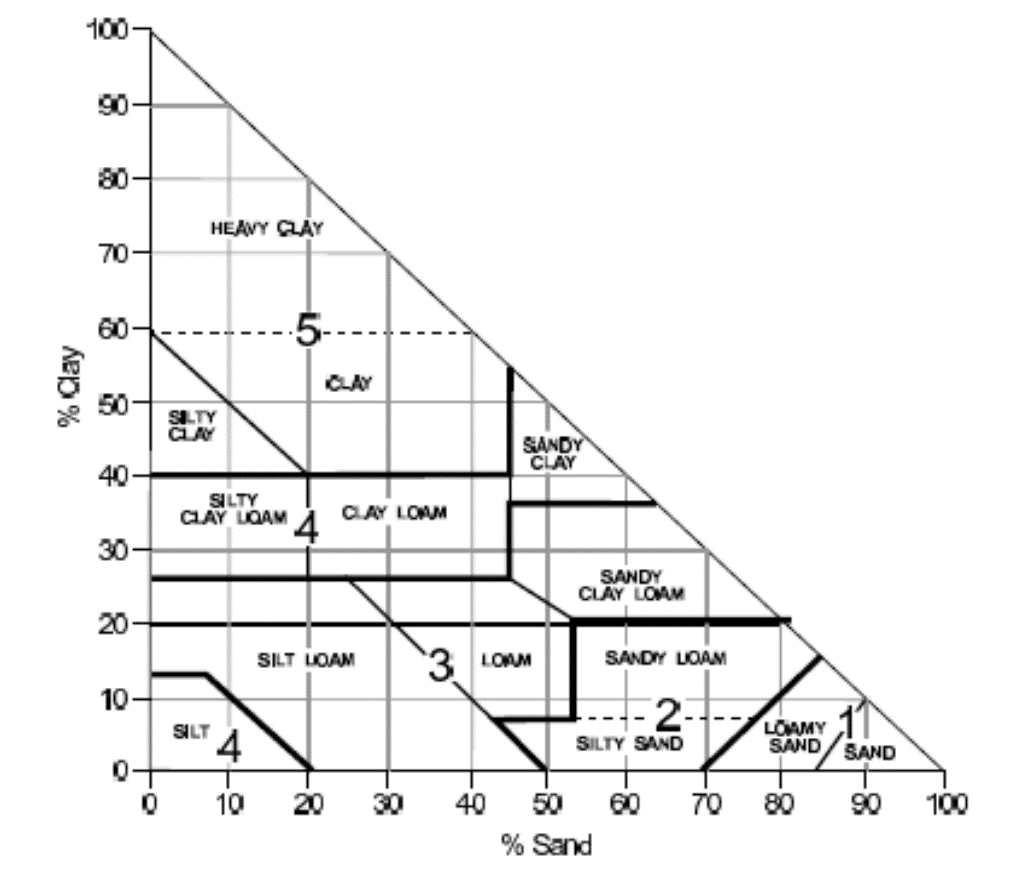
*

*Figure S3b: Permeability code based on textural classification (Ontario Center for Soil Resource Evaluation, 1993)*

1. **Determination of Organic Matter Content of the Soil Samples**

This test is performed using the (Walkey-Black Method) to determine the organic content of soils. The organic content is the ratio, expressed as a percentage, of the mass of organic matter in a given mass of soil to the mass of the dry soil solids. Organic matter influences many of the physical, chemical and biological properties of soils. Some of the properties influenced by organic matter include soil structure, soil compressibility and shear strength. In addition, it also affects the water holding capacity, nutrient contributions, biological activity, and water and air infiltration rates. The organic matter content of the soil samples were analyzed in the Sanitary Labouratory, Department of Civil Engineering, Abubakar Tafawa Balewa University, Bauchi.

**SUPPLEMENTARY FILE 4**


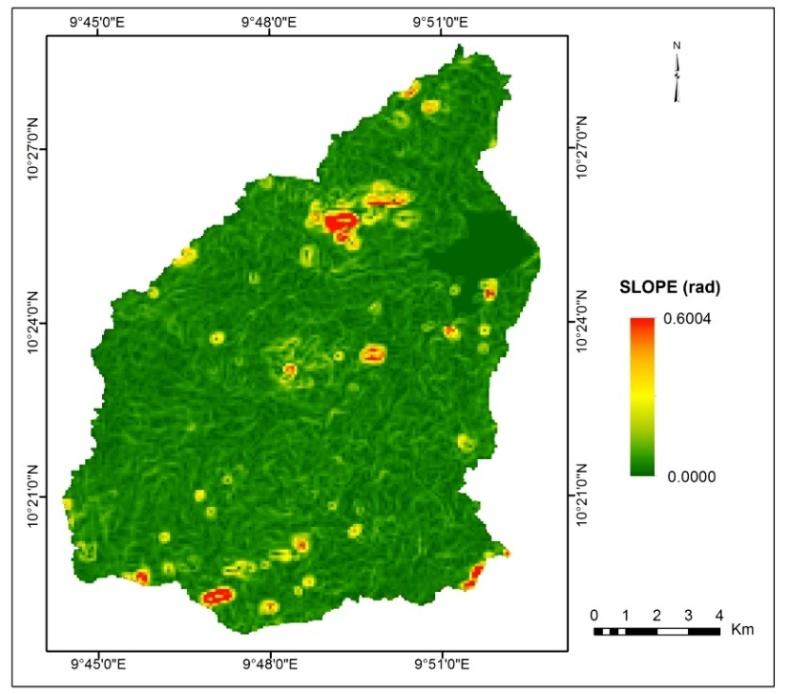


*Figure S4a: Landuse Coefficient of Runoff Flow (𝑎i Map)*


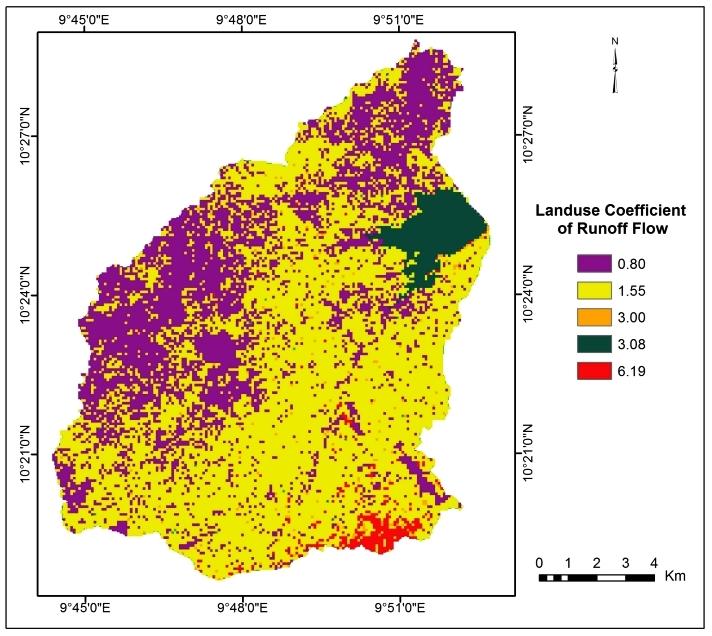


*Figure S4b: Slope (radian) of Gubi Dam Watershed (𝑆i Map)*
